# Temporary Cohabitation: The Metastable Phase Au_4_Si

**DOI:** 10.1021/jacs.2c10306

**Published:** 2022-11-14

**Authors:** Julia-Maria Hübner, Brenna C. Bierman, Reine Wallenberg, Daniel C. Fredrickson

**Affiliations:** † Centre for Analysis and Synthesis, Lund University, Naturvetarvägen 14, 221 00 Lund, Sweden; ‡ Department of Chemistry, University of Wisconsin−Madison, 1101 University Avenue, Madison, Wisconsin 53726, United States

## Abstract

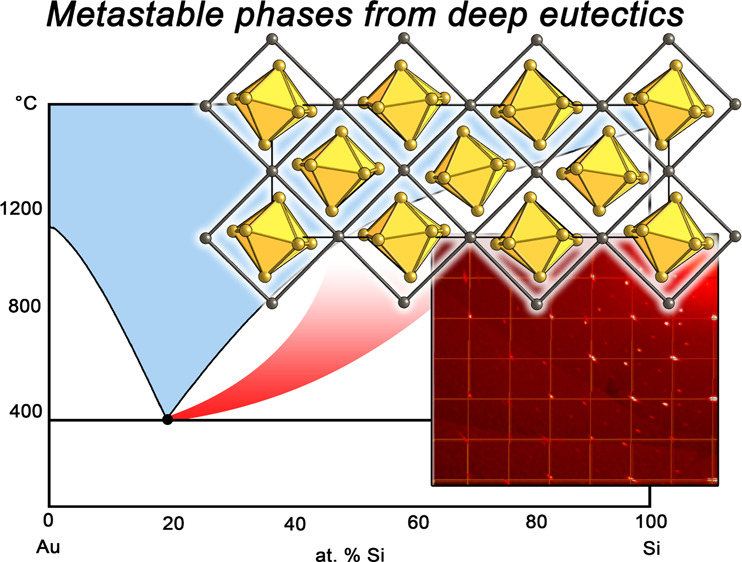

The prediction, identification, and characterization of phases away from equilibrium conditions remain difficult challenges for material science. Herein, we demonstrate how systems whose phase diagrams contain deeply incising eutectics can offer opportunities to address these challenges. We report the synthesis of a new compound in the Au–Si system, a textbook example of a system with a deep eutectic. Au_4_Si crystallizes in a complex √18×√2×1 superstructure of the PtHg_4_ type, based on the distortion of vertex-sharing Si@Au_8_ cubes into bisdisphenoids. Au_4_Si decomposes upon heating and at room temperature even in high vacuum, highlighting its metastability. Electronic structure analysis reveals a pseudogap at the Fermi energy, which is enhanced by the superstructure through the relief of Au–Au antibonding interactions. The pseudogap is associated with a Zintl-type bonding scheme, which can be extended to the locally ordered liquid. These results highlight the potential for metastable phases to form in deep eutectics that preserve the local structures of the liquid.

In the gold–silicon system, the eutectic temperature *T*_e_ = 363(3) °C at ∼20 at. % Si is substantially lower than the melting points of the constituent elements.^1,2^ The low melting point has been exploited for the catalyzed growth of Si nanowires,^3−6^ layering of Au–Si atoms in the liquid when in contact with a crystalline substrate,^7^ and metallic glass formation.^8^ Such deeply incising eutectics can indicate a high dispersion and strong local order in the melt at the expense of the possible formation of solids.^9,10^ The possibility of supercooling of the eutectic gold–silicon liquid^7^ and the striking observation of monolayer crystallization at the liquid surface several degrees Celsius above the equilibrium eutectic point^11^ are suggestive of strong ordering phenomena in the melt.^12^ The existence of a crystalline phase in the Au–Si system has been suspected since the 1930s.^13−18^ Herein, we present the synthesis of the compound Au_4_Si, whose local structural features hint that geometrical motifs predefined in the melt have been preserved as bulk samples comprising single crystals suitable for diffraction experiments. Au_4_Si represents a remarkable example of a metastable phase in intermetallic chemistry, a domain where, unlike molecular and biochemical systems,^19^ thermodynamics generally dictates the products of a synthesis.

Single-phase samples of Au_4_Si were synthesized by arc melting stoichiometric combinations of the elements. High-quality single crystals (Figure 1) could be obtained from carefully crushed samples. Mechanical stress leads to partial decomposition, and further manipulation of single crystals, e.g., by cutting, causes a reduced crystal quality. As-cast samples of Au_4_Si are brittle and possess a silver color that becomes golden within a few days in air. The process also occurs in the absence of oxygen or moisture but is considerably slower. An as-cast sample sealed under vacuum in a fused silica ampule in 2015 is now showing signs of decomposition, taking on a golden hue in patches.

**Figure 1:**
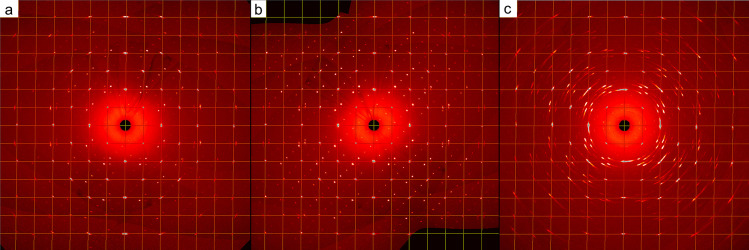
Diffraction pattern of Au_4_Si. Reciprocal lattice reconstructions of (a) (0*kl*), (b) (*h*0*l*), and (c) (*hk*0) layers illustrate the pattern of main reflections and intensity distributions of superstructure reflections. The crystal exhibits unbalanced twinning with superstructure peaks along the face diagonals of the pseudocubic basic cell in reciprocal space.

For a deeper analysis of the decomposition process, we collected powder X-ray diffraction patterns of a bulk sample of Au_4_Si over time. The decomposition into Au and Si in air (Figure 2, top) can be described with the formula for decay *A*(*t*) = *A*_0_·e^–λ·*t*^,^20^ corresponding to a half-life of 17.3 days. The peak widths (FWHM) of Au_4_Si (Figure 2, bottom) exhibit a profound change within the first 24 h. This observation can be assigned to a disintegration of the most strained particles of Au_4_Si and/or a healing process over time similar to the one observed for Au microparticles.^21^ In contrast, the gold particles arising as a decomposition product undergo a substantial increase in peak width within the first 95 h. Single crystals of Au_4_Si isolated from the original matrix are relatively short-lived, exhibiting powder rings or turning amorphous within a few hours (Figure 1c).

**Figure 2:**
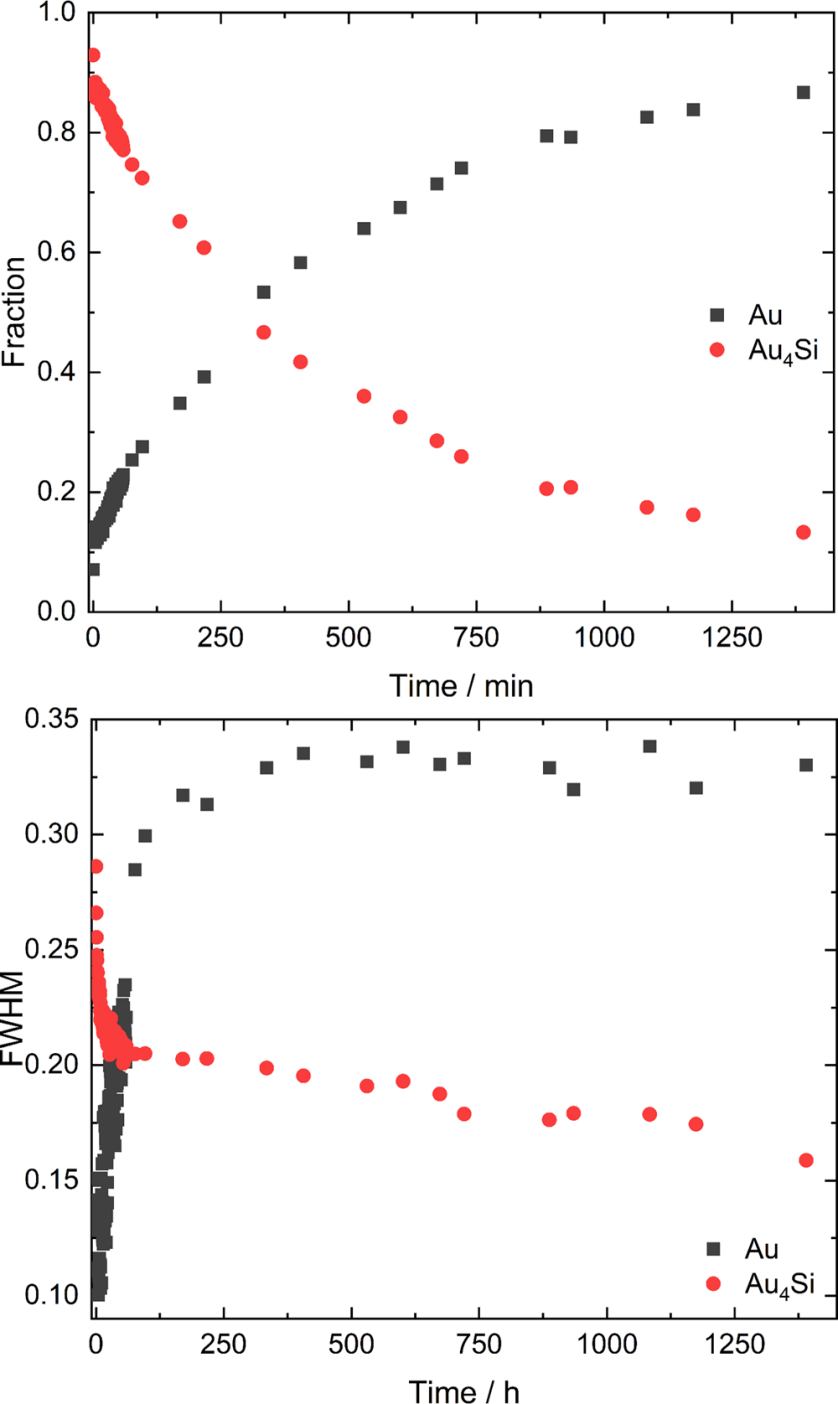
Phase fractions (top) and peak widths (FWHM, bottom) of Au_4_Si and Au versus time.

Upon heating, the DSC curve shows a signal with *T*_onset_ = 495(10) K (Figure S1). In agreement with ref (22), this signal corresponds to the start of decomposition of Au_4_Si and formation of Au, although in situ heating experiments with synchrotron radiation (DESY, Hamburg, Germany) revealed that very high-quality crystals of Au_4_Si withstand temperatures of up to 573 K for ∼4 min. At *T*_onset_ = 635(10) K, an endothermic effect with a corresponding signal in the cooling curve is observed, in line with the *T*_e_ of the eutectic mixture.^23^ No signal denoting reformation of Au_4_Si was detected upon cooling.

The specific heat *C*_*p*_(*T*) of Au_4_Si (Figure S6) shows a complex trend with a transition at *T*_onset_ = 260 K. However, DSC measurements in the temperature range from 185 to 310 K do not denote a phase transition (Figure S1). The Debye temperature θ_D_,^24,25^ providing a measure of the phonon contribution to the specific heat, amounts to 254 K at low temperatures (2 < *T* < 210 K), being in good agreement with the value estimated from Neumann–Kopp’s rule for eutectic mixtures^26^ (259 at 0 K),^27^ but lowers to 210 K at higher temperatures (200 < *T* < 400 K) (Table S4), which is in good agreement with the one previously reported for a mixture Au_81.4_Si_18.6_ (θ_D_ = 220 K).^23^

For structure determination, single-crystal diffraction experiments were conducted at room temperature. From the main reflections an average, *I*-centered, cubic cell can be identified with *a* ≈ 5.5 Å. In addition, superstructure reflections forming cuboctahedra around the loci of the systematically extinct reflections of the basic structure can be observed (Figure 1). The intensity of these additional reflections is unbalanced with respect to cubic symmetry and shows a slight preference in certain directions (Figure 1,a,b), pointing toward a deviation from a cubic symmetry.

Assuming that the superstructure is, in fact, unidirectional along one face diagonal to the reciprocal cubic unit cell, the full crystal structure was solved using the charge-flipping method.^28,29^ It belongs to the space group *C*2*ce* (standard setting *Aea*2) with *a* = 5.5486(3) Å, *b* = 23.6323(7) Å, and *c* = 7.8828(2) Å (Table S1). Six directions of orthorhombic symmetry form a pseudocubic arrangement, leading to six possible twin directions (excluding the inversion twins). The introduction and refinement of the corresponding twin fractions led to a significant improvement of the fit from *R* = 0.1645 to *R* = 0.0507 (Table S1) for a model with five unequal contributions.

The refined structure constitutes a √18×√2×1 superstructure of a distorted PtHg_4_-type arrangement, whose symmetry can be accounted for with a Bärnighausen tree (Figure S3). Six independent Au and two Si fully occupied positions are observed (Table S2). The resulting composition, Au_4_Si, is in sound agreement with TEM/EDXS measurements, which yield a ratio Au:Si of 3.96(5):1.

The structure comprises corner-sharing Au bisdisphenoids (Figure 3b) centered by Si (CN = 8), which are tilted alternatingly (Figure 3a) similar to tilt modes observed, for example, in perovskites.^30^ Two layers of bisdisphenoids centered by Si1 (A) are followed by one with Si2-containing bisdisphenoids (B) in the [010] direction in an ...AABAA... manner. Between the bisdispheniods, a space-filling arrangement is completed by two types of empty voids in the shape of stellae quadrangulae (Figure 3b).^31−35^

**Figure 3:**
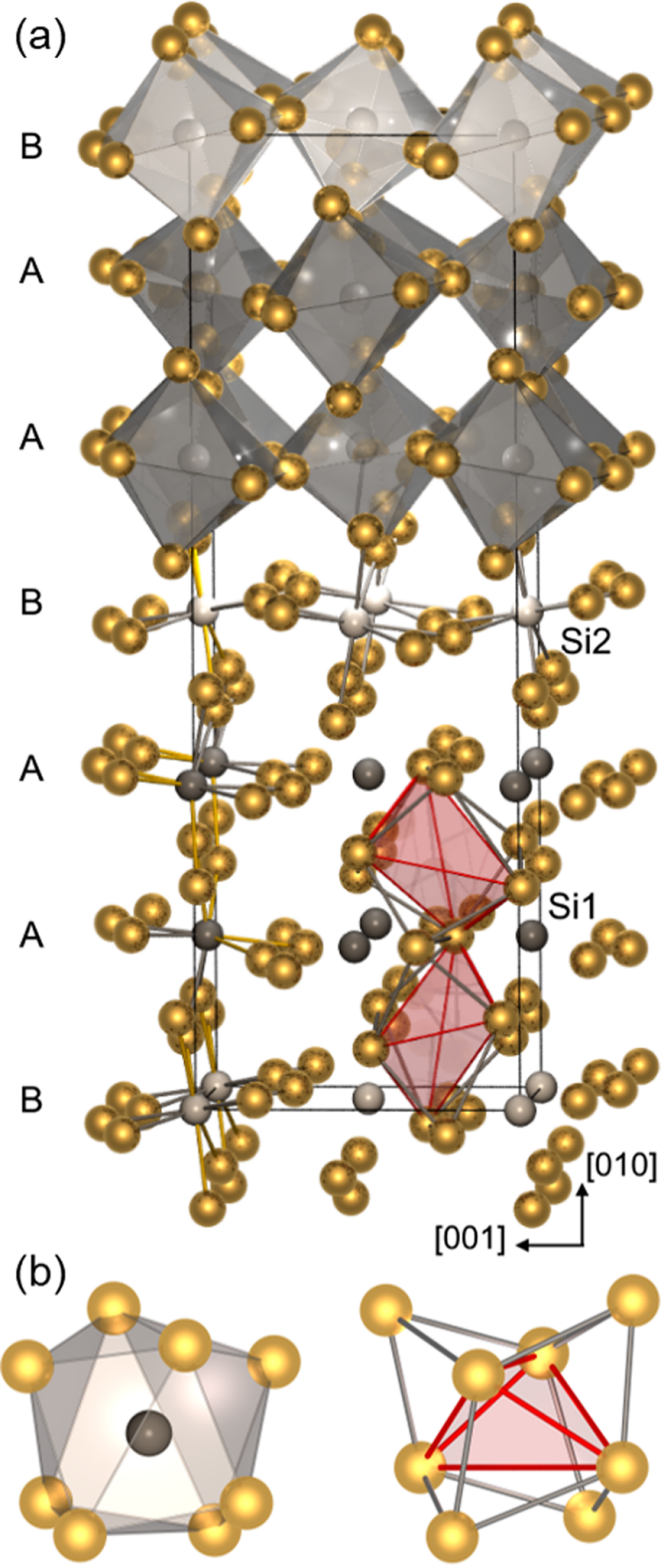
(a) Crystal structure of Au_4_Si. Labels A and B refer to slabs of Si@Au_8_ bisdisphenoids centered by Si1 (dark gray) and Si2 (light gray), respectively. Shorter Au–Si distances are indicated in yellow. (b) Motifs in Au_4_Si: bisdisphenoid around Si1 (left) and stella quadrangula around the interstices (right, red in (a)).

The occurrence of corner-sharing bisdisphenoids as the dominating polyhedron points toward initial SiAu_8/2_ clusters in the melt in-line with the occurrence of crystalline surface phases in the liquid state of Au–Si eutectic mixtures.^11^ Indeed, each polyhedron comprises four shorter and four longer Au–Si distances, all within the range of 2.48 to 2.58 Å (Figure S2). The shorter distances are similar to nearest-neighbor Au–Si correlations of ∼2.4 Å in molten Au–Si eutectic mixtures.^36^

The Au–Au distances of Au_4_Si (Table S3) are, on average, very similar to those in elemental gold and gold-rich binaries containing p-block elements (Figure S4).^37−48^

To understand the unique bonding and structural features of Au_4_Si, we carried out electronic structure calculations with DFT. The electronic density of states (DOS) distribution (Figure 4) for the observed Au_4_Si structure exhibits a pseudogap at the Fermi energy (*E*_F_), hinting that the phase has a nearly optimized bonding at its sp valence electron count of 8 per formula unit, which is consistent with the brittleness and diamagnetic behavior observed for the compound (Figure S5).

**Figure 4:**
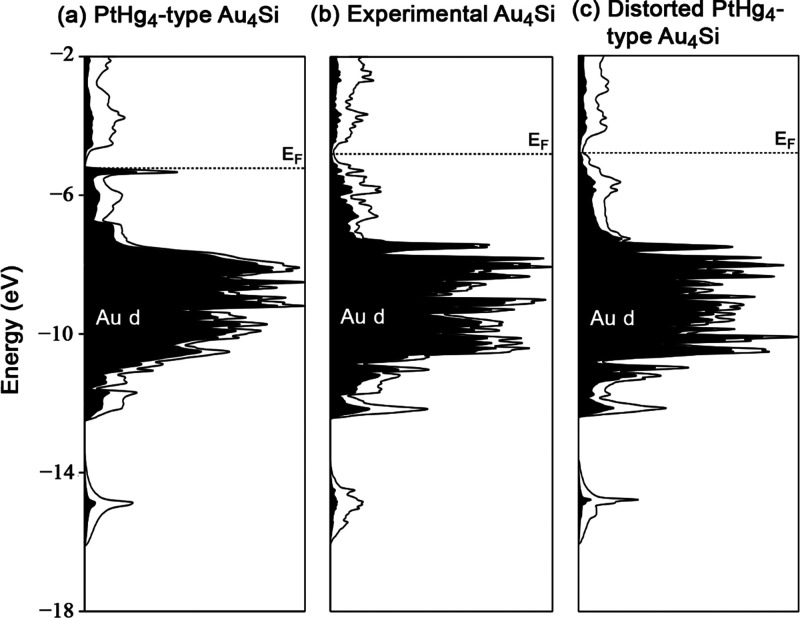
DFT electronic DOS distributions for three models of Au_4_Si. (a) A hypothetical PtHg_4_-type form. (b) The experimentally observed superstructure. (c) A distorted version of the PtHg_4_-type form, in which the cubes become bisdisphenoids without disrupting the translational symmetry. Au d contributions are black shaded.

The origin of this pseudogap can be traced through a series of calculations on related structures. We begin in Figure 4a, with a hypothetical PtHg_4_-type form of Au_4_Si. Here the *E*_F_ lies right at the top of a sharp peak of states at the bottom of a DOS minimum, suggesting that the phase has a nearly optimized electron count, but with the occupied levels having a high weight at the upper end. Upon moving to the experimental structure, the DOS minimum widens (Figure 4b), and the peak just below *E*_F_ largely disperses to lower energies. As such, the formation of the superstructure appears to be driven by the stabilization of these frontier states.

To test whether this stabilization stems from local changes in the coordination environment or the specific periodicity of the superstructure, we calculated a DOS distribution for a distorted version of the PtHg_4_-type structure in which the Si@Au_8_ cubes are replaced with bisdisphenoids, while keeping the original translational symmetry (Figure 4c). Here, the states just below the *E*_F_ in the PtHg_4_ are similarly stabilized, highlighting the importance of the cube → bisdisphenoid transformation.

The origin of this effect is more clearly seen in the band structures (Figure 5). In the band structure of the PtHg_4_ type, the *E*_F_ is accompanied by a flat and degenerate set of bands just below it for many of the paths shown, consistent with the peak in the DOS distribution. Upon moving to the distorted version of the PtHg_4_ type, the opening of the pseudogap coincides with the lowering of this set of bands in energy relative to the *E*_F_.

**Figure 5:**
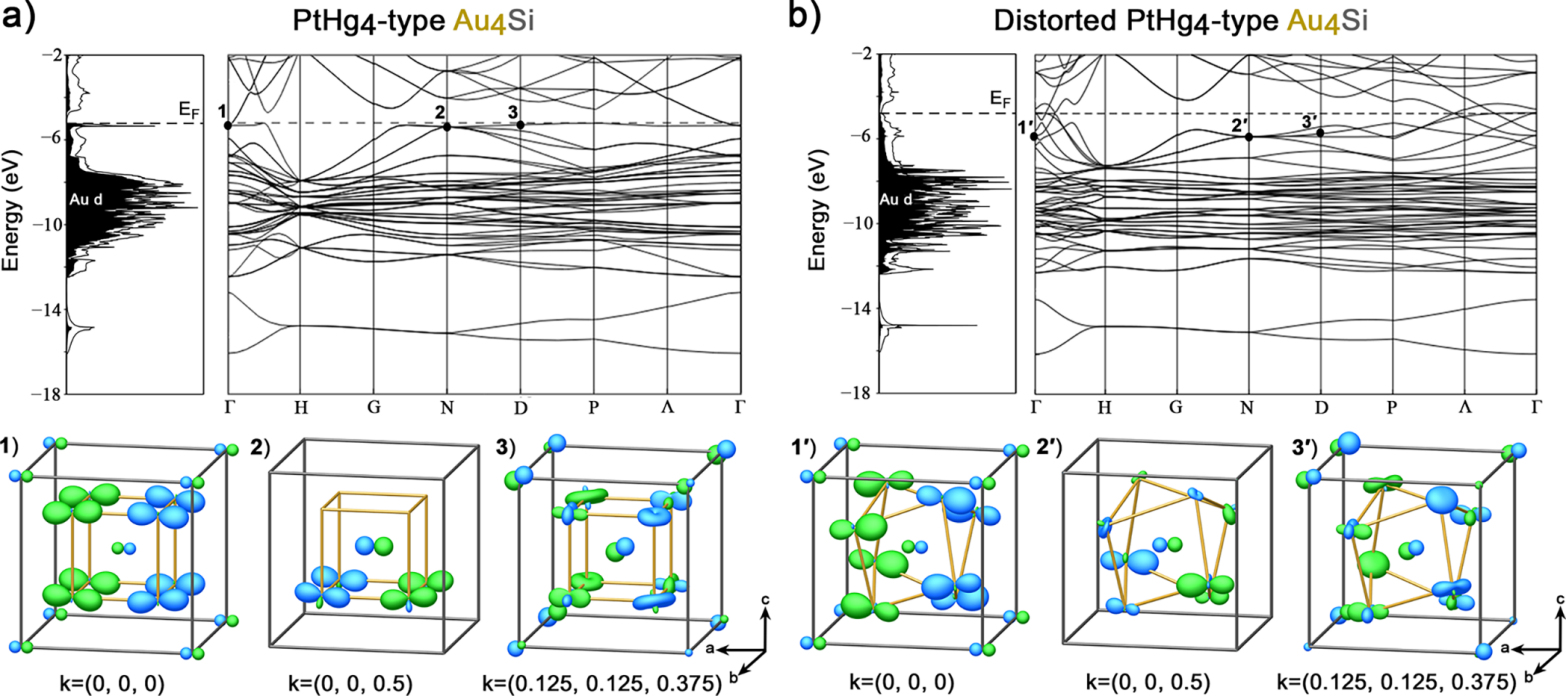
Comparison of DOS distributions, band structures, and selected crystal orbitals for (a) a PtHg_4_-type version of Au_4_Si and (b) a distorted variant in which the Si@Au_8_ cubes are replaced with bisdisphenoids within the same *I*-centered cell. The special points and *k*-vectors refer to the reciprocal lattice for the conventional cell of the PtHg_4_ type. For simplicity, only the real component of each crystal orbital is shown.

In the lower part of Figures 5a,b, we explore the nature of these bands by plotting pictures of the crystal orbitals at three selected *k*-points: Γ (0,0,0), N (0,0,1/2), and D (1/8,1/8,3/8). For the PtHg_4_ type, the crystal orbitals involve the Si p orbitals overlapping in a bonding fashion with Au orbitals derived chiefly from the Au d_*z*^2^_, d_*x*^2^–*y*^2^_, and s. This is most clear for the Γ point, in which each Au atom has a pair of lobes pointing in opposite directions with the same phase, resembling a d_*z*^2^_ orbital without its torus. The Au d_*z*^2^_, d_*x*^2^–*y*^2^_, and s can in fact be hybridized to make three such sd^2^ functions oriented along the *x*, *y*, and *z* axes, consistent with the 3-fold degeneracy of this band at Γ. The corresponding crystal orbitals at points D and N show similar combinations of d_*z*^2^_, d_*x*^2^–*y*^2^_, and s orbitals on each Au atom, but with more complicated shapes.

The relatively high energy of these orbitals at Γ can be understood by noting that, in bonding to opposite lobes of the Si p orbitals, neighboring Au atoms are antibonding with each other. This effect is particularly severe, as the arrangement of the Au atoms on a primitive cubic network means that the apexes of the lobes on neighboring atoms align directly with each other. Similar combinations of Si–Au bonding and Au–Au antibonding persist at the *k*-points D and N.

The parallel alignment of the Au lobes in these crystal orbitals yields a simple picture for the stabilizing role of the distortion of the cubes to bisdisphenoids. When introducing this distortion (Figure 5b), these bands drop in energy as the arrangement of the Au atoms into linear chains is broken in all three directions, and the antibonding overlap between the Au atoms decreases. Au–Si bonding can now occur without implying strong Au–Au antibonding, allowing for for better stabilization of the states just below the *E*_F_.

The origin of this pseudogap has broader implications for the Au–Si system. As is shown with reversed approximation molecular orbital (raMO) analysis^49^ (Figure S11), the occupied crystal orbitals of Au_4_Si can be interpreted in terms of the Zintl-like configuration [Au^+^]_4_[Si^4–^]. The raMO functions reveal strong covalency in the Au–Si interactions, which balances the extreme polarity evoked by the formal charges in this picture. Note that this bonding scheme depends more on the 4:1 Au:Si composition than the geometrical details; it would be expected to apply as well to the eutectic liquid (perhaps contributing to its low *T*_e_), with its local rather than long-range order. This highlights the ease with which Au_4_Si could form from preorganized units in the melt upon cooling past the eutectic point.

Altogether, we have presented the synthesis of the metastable binary phase Au_4_Si near the eutectic point in the Au–Si system. Structural characterization revealed a unique atomic arrangement related to the PtHg_4_ type that optimizes the balance of Au–Si and Au–Au interactions. The formation conditions of Au_4_Si and the underlying electronic principles open a window into the rich structural chemistry of deep eutectic systems and the potential for the discovery of new metastable phases.

## Supporting Information Available

Synthesis, materials, characterization, and computational details, and additional results including crystallographic data, thermal analysis, band structures, raMO functions, DOS curves calculated with and without spin–orbit coupling, and physical property measurements (PDF)
