# Supplementary material for: Temporary Cohabitation: The Metastable Phase Au4Si
Source: J Am Chem Soc. 2022 Nov 14;144(46):21016–21. doi: 10.1021/jacs.2c10306 (PMC9706559; doi:10.1021/jacs.2c10306)
Supplement: Supplementary file 1 — ja2c10306_si_001.pdf [file ja2c10306_si_001.pdf]

SUPPORTING INFORMATION

Temporary cohabitation: the metastable phase Au_4Si

Julia-Maria Hübner[1,*], Brenna C. Bierman[2], Reine Wallenberg,[1] and Daniel C. Fredrickson[2]

[1] Centre for Analysis and Synthesis, Lund University, Naturvetarvägen 14, 22100 Lund, Sweden.
[2] Department of Chemistry, University of Wisconsin-Madison, 1101 University Avenue, Madison, Wisconsin 53726, United States.

1. Preparation
2. Thermal analysis
3. Crystallographic data from single-crystal X-ray diffraction data
4. Crystal chemical and symmetry relations
5. Transmission electron microscopy and energy-dispersive X-ray spectroscopy
6. Physical properties
7. Computations
8. References

1. Preparation

Sample handling, except for diffraction experiments, and storage were conducted in an argon-filled glove box (MBraun, H_2O<0.1 ppm, and O_2<0.6 ppm). The samples were synthesized from pure Au and Si using an arc melter (Edmund Bühler GmbH, MAM-1) in argon atmosphere. All sample transfer was conducted in argon-filled containers.

2. Thermal analysis

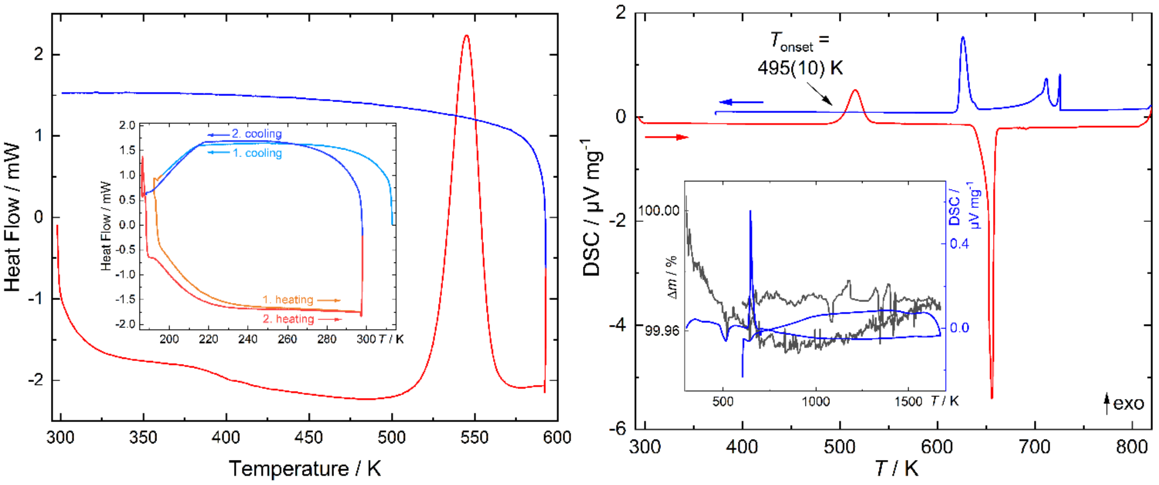

Figure S1. (left) DSC measurement of Au_4Si between 300 and 590 K, and (inset) between 185 and 310 K in nitrogen atmosphere. (right) DSC measurement of Au_4Si between 290 and 820 K, and DSC/TG experiment between 300 and 1675 K in argon atmosphere.

Differential Scanning Calorimetry (DSC) was performed in a NETZSCH DSC 214 with a concave Al pan and a pierced lid between 20 and 550°C and heating and cooling rates of 10 K min^{-1} (sample mass 111.6 mg) in argon atmosphere and between -50 and 300°C in a DSC Q2000 device with heating and cooling rates of 10 K min^{-1} (sample mass 25.568 mg) and nitrogen atmosphere in an Al pan and lid. Simultaneous Thermal Analysis with simultaneous application of Thermogravimetry (TG) and DSC was performed in a NETZSCH STA 449F3 with an Al_2O_3 DSC/TG pan between 30 and 1400°C with a heating rate of 10 and a cooling rate of 40 K min^{-1} (sample mass 164.1 mg) in argon atmosphere.

DSC measurements of Au_4Si in argon and nitrogen atmosphere (Figure S1) reveal an exothermic effect with T_{onset} = 495(10) K, which is in sound agreement with findings in [1]. This effect could denote a phase transition to a high-temperature phase of Au_4Si or the recrystallization of elemental Au. Since the quenching of this possible high-temperature phase was not achieved, in-situ temperature-dependent X-ray diffraction experiments were conducted (see paragraph 3). A corresponding signal upon cooling, as well as in the second heating cycle that could denote the reformation of Au_4Si is not observed. Upon further heating, an endothermic effect at T_{onset} =

635(10) K with a corresponding signal in the cooling curve is observed, which is in agreement with the melting point T_m of the eutectic mixture [2]. Further effects upon cooling are assigned to reactions with the Al crucible. In STA measurements (Al_2O_3 crucible), TG shows a very small weight loss during heating (<99.95 %), while the signals in DSC accord with the ones observed during the further DSC experiment (Al crucible), while no crucible reaction occurred (Figure S1, right, inset).

Powder X-ray diffraction experiments after the DSC experiment between 290 and 820 K show elemental Au and Si and the phases Au_4Al [3] and Al_3Au_8 [4] denoting a reaction with the crucible, whereas after the DSC/TG experiment between 300 and 1675 K only elemental Au and Si, as well as the unreacted crucible material Al_2O_3 were observed.

3. Crystallographic data

Powder X-ray diffraction experiments were executed (Stoe Stadi MP, Mythen 1k detector, Cu-K alpha radiation, λ=1.54178 Å) in transmission mode. Data analysis was done with the Stoe WinXPOW software version 3.06.

Single crystal diffraction experiments were performed with an Xcalibur 3 single crystal X-ray diffractometer (Rigaku, CCD plate detector, graphite monochromator, Mo Kα radiation, λ=0.71073 Å) and at the beamline Cristal (Synchrotron Soleil, France, λ=0.58173 Å). Temperature-dependent single crystal diffraction experiments were executed at beamline P24 (DESY, Hamburg, Germany, λ= 0.50000 Å). Data integration, structure solution, and refinements were conducted with the programs CrysAlisPro [5], Superflip [6], and JANA2006 [7].

Table S1. Single Crystal XRD data for Au_4Si. investigated with synchrotron radiation (293 K). Further details on the crystal structure investigations can be obtained from the FachinformationszentrumKarlsruhe, 76344 Eggenstein-Leopoldshafen, Germany (email: crysdata@fiz-karlsruhe.de, http:///www.fiz-karlsruhe.de/request_for_deposited_data.html) on quoting the depository numbers CSD-2204716.

Composition	Au_4Si
Space group, Pearson symbol	*C*2*ce* (No. 41), *oS*40
Unit cell parameters	
a [Å]	5.5486(3)
b [Å]	23.6323(7)
c [Å]	7.8828(2)
V [Å^3]	1033.64(7)
Formula units *Z*	8
Diffractometer	Xcalibur Atlas, CCD detector, Synchrotron radiation λ = 0.58173 Å
Reflections collected / independent within $I > 3\sigma(I)$	20883 / 3004
Fourier difference ρ_{min}/ρ_{max} (electrons/Å^3)	7.37/-7.09
Measurement range	$-9 \leq h \leq 9$, $-42 \leq k \leq 42$, $-13 \leq l \leq 13$
Residuals and GOF	*R* = 0.0507, *wR* = 0.0603, GOF = 2.62

Table S2. Position and displacement parameters for Au_4Si.

Atom	Site	*a* / x	*b* / y	*c* / z	U_{anis}*	Occ.
Au1	8*b*	0.1514(3)	0.23255(4)	0.9811(1)	0.0218(2)	1.0
Au2	8*b*	0.8409(3)	0.17516(3)	0.2053(1)	0.0147(2)	1.0
Au3	8*b*	0.8473(3)	0.15090(5)	0.7973(1)	0.0212(2)	1.0
Au4	8*b*	0.1633(2)	0.09759(3)	0.0456(1)	0.0162(2)	1.0
Au5	8*b*	0.6587(2)	0.06639(3)	0.0517(1)	0.0153(2)	1.0
Au6	8*b*	0.3447(3)	0.01805(3)	0.7956(1)	0.0151(2)	1.0
Si1	8*b*	0.003(2)	0.1674(2)	0.5071(9)	0.016(2)	1.0
Si2	4*a*	0.004(2)	0	0	0.015(2)	1.0

* Au1: U_{11}=0.0166(4), U_{22}=0.0128(3), U_{33}=0.0359(5), U_{12}=0.0020(3), U_{13}=0.0076(4), U_{23}=0.0083(3); Au2: U_{11}=0.0116(3), U_{22}=0.0199(3), U_{33}=0.0125(3), U_{12}= 0.0009(3), U_{13}=-0.0004(3), U_{23}= 0.0042(3); Au3:

U_{11}=0.0139(3), U_{22}=0.0371(4), U_{33}=0.0126(3), U_{12}=0.0017(4) , U_{13}= 0.0021(3), U_{23}= 0.0038(3); Au4: U_{11}=0.0120(3), U_{22}=0.0123(3), U_{33}=0.0242(4), U_{12}= -0.0008(3), U_{13}= 0.0004(4), U_{23}=-0.0050(3); Au5: U_{11}= 0.0116(3), U_{22}=0.0112(2), U_{33}=0.0232(4), U_{12}= 0.0011(3), U_{13}= -0.0013(4), U_{23}=0.0009(3); Au6: U_{11}=0.0122(3), U_{22}=0.0216(3), U_{33}=0.0116(3) , U_{12}= 0.0002(3) , U_{13}=0.0014(3) , U_{23}=-0.0033(3); Si1: U_{11}=0.015(3), U_{22}=0.016(3), U_{33}=0.017(4) , U_{12}=-0.001(2) , U_{13}=-0.004(2) , U_{23}=0.001(2);Si2: U_{11}= U_{22}=U_{33}=0.015(4), U_{12}=U_{13}=0, U_{23}=-0.001(3)

Table S3. Selected interatomic distances in Au_4Si.

Atom	Distance / Å	Atom	Distance / Å
Au1 – 2 Au1	2.9097(5)	Au1 – Si1	2.489(9)
Au1 – Au2	2.816(1)	Au1 – Si1	2.511(6)
Au1 – Au2	2.832(1)	Au2 – Si1	2.551(7)
Au1 – Au2	3.010(1)	Au2 – Si1	2.518(9)
Au1 – Au3	2.944(1)	Au3 – Si1	2.477(7)
Au2 – Au1	2.816(1)	Au3 – Si1	2.484(9)
Au2 – Au1	2.832(1)	Au4 – Si1	2.541(9)
Au2 – Au1	3.010(1)	Au4 – 2 Si2	2.497(4)
Au2 – 2 Au2	2.863(2)	Au5 – Si1	2.508(6)
Au2 – Au4	2.854(1)	Au5 – 2 Si2	2.51(1)
Au2 – Au4	2.862(1)	Au6 – 2 Si2	2.522(9)
Au2 – Au5	3.016(1)	Au6 – 2 Si2	2.528(4)
Au3 – Au1	2.944(1)	Si1 – Au1	2.489(9)
Au3 – 2 Au3	2.873(2)	Si1 – Au1	2.511(6)
Au3 – Au4	2.914(2)	Si1 – Au2	2.551(7)
Au3 – Au5	3.018(1)	Si1 – Au2	2.518(9)
Au4 – Au2	2.854(1)	Si1 – Au3	2.477(7)
Au4 – Au2	2.862(1)	Si1 – Au3	2.484(9)
Au4 – Au3	2.914(2)	Si1 – Au4	2.541(9)
Au4 – Au5	2.846(2)	Si1 – Au5	2.508(6)
Au4 – Au5	2.896(2)	Si2 – 2 Au4	2.497(4)
Au4 – Au6	2.903(1)	Si2 – 2 Au5	2.51(1)
Au5 – Au2	3.016(1)	Si2 – 2 Au6	2.522(9)
Au5 – Au3	3.018(1)	Si2 – 2 Au6	2.528(4)
Au5 – Au4	2.846(2)		
Au5 – Au4	2.896(2)		
Au5 – Au6	2.901(2)		
Au5 – Au6	2.909(2)		
Au5 – Au6	2.957(1)		
Au6 – Au4	2.903(1)		
Au6 – Au5	2.901(2)		
Au6 – Au5	2.909(2)		
Au6 – Au5	2.957(1)		
Au6 – 2 Au6	2.866(2)		

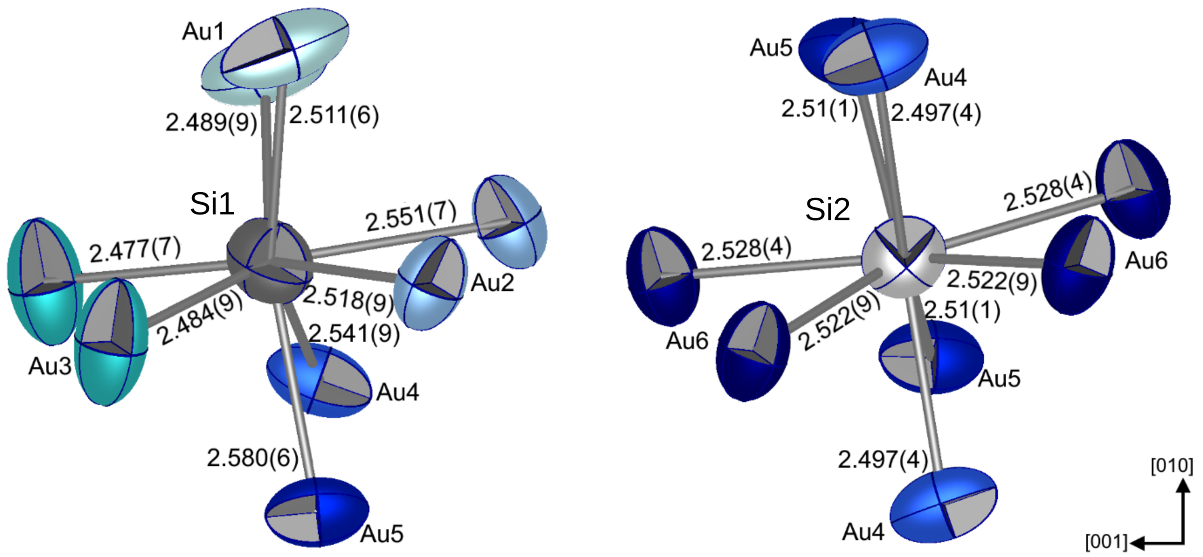

Figure S2. Interatomic distances of Si1 and Si2 in Å.

Temperature-dependent diffraction experiments on single crystals in air and sealed in capillaries under argon atmosphere revealed that the exothermic effect with T_{onset} = 495(10) K visible during DSC and STA experiments is assigned to a partial decomposition into polycrystalline Au upon heating. Nevertheless, the Au_4Si structure is also stable at higher temperatures and was detected for ~4 min at the maximum investigated temperature of 573 K.

4. Crystal chemical and symmetry relations

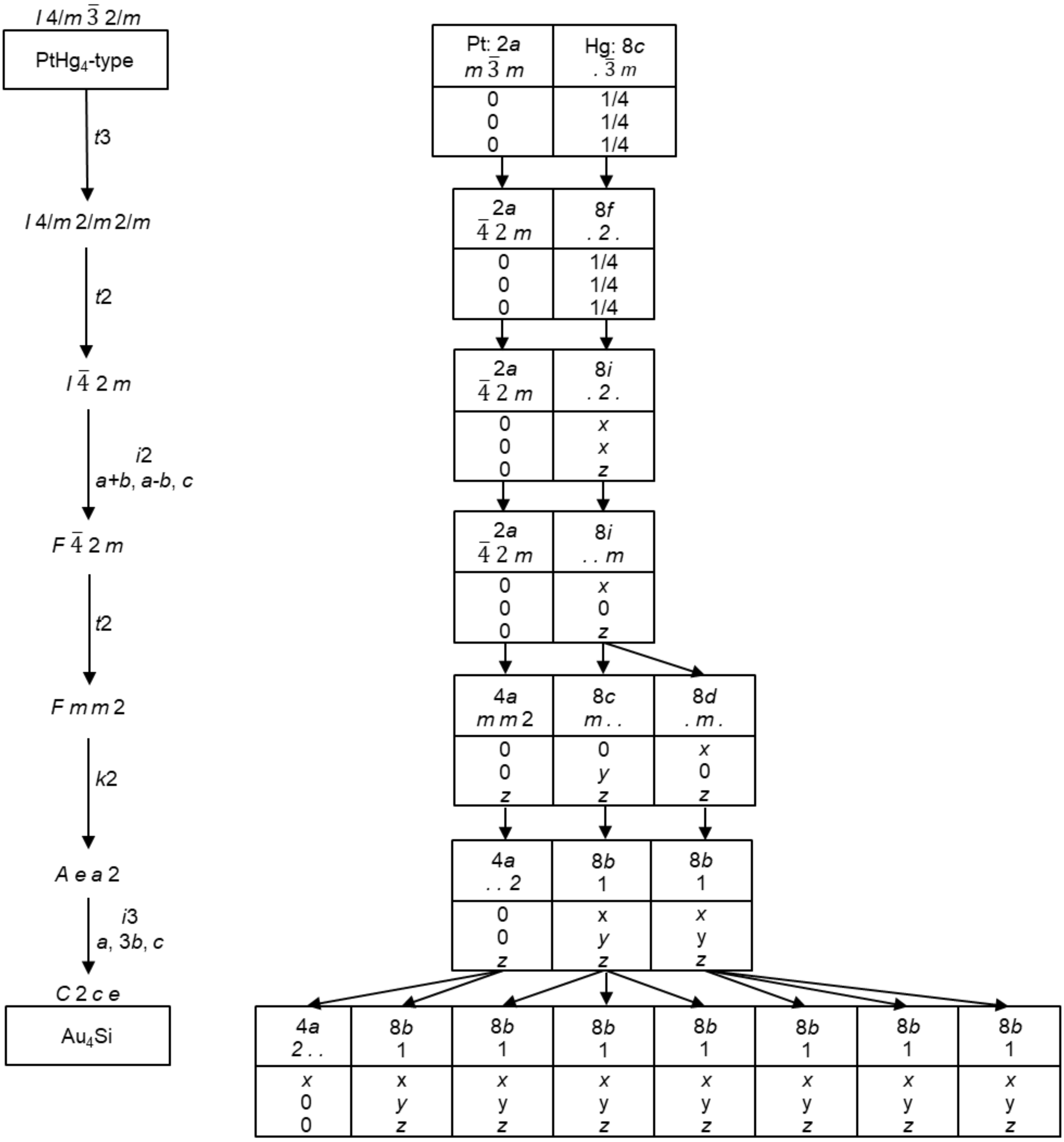

Figure S3. Bärnighausen tree showing the relation between the aristotype $PtHg_4$ and the hettotype Au_4Si.

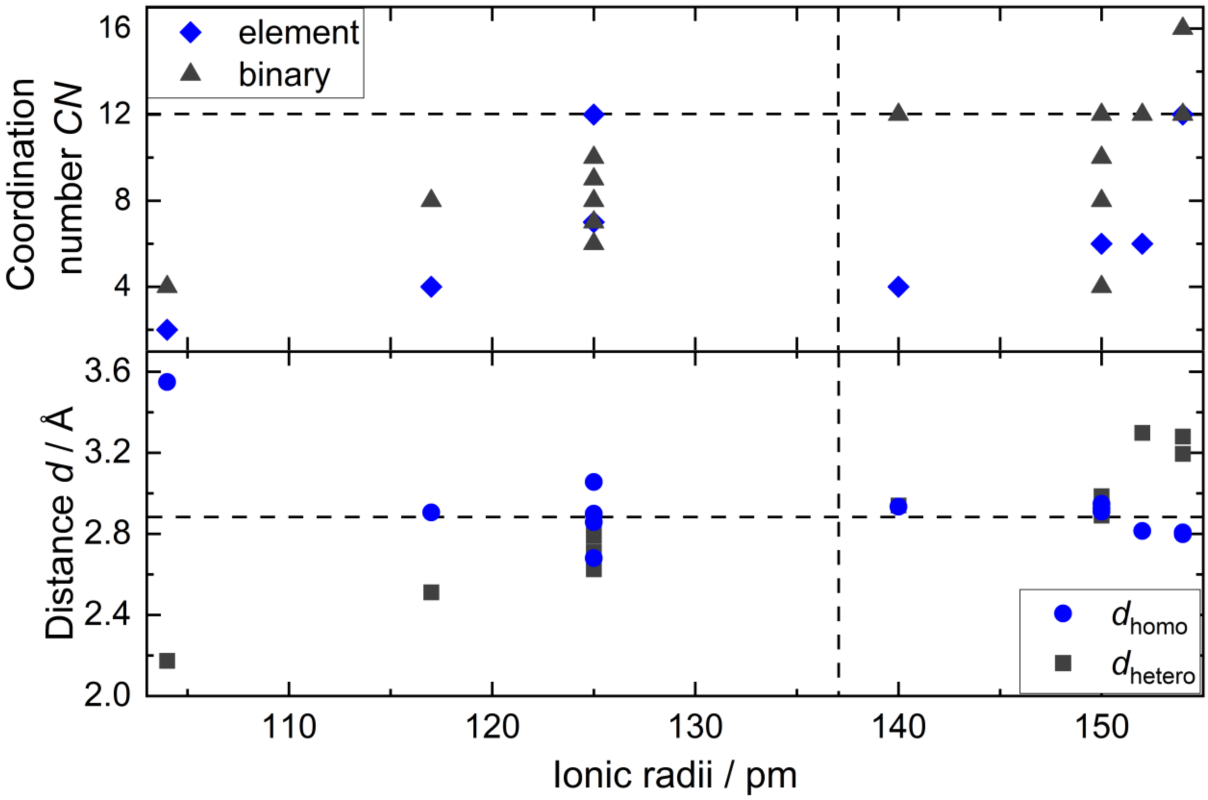

Figure S4. (top) Coordination numbers CN vs. ionic radii of gold-rich binaries comprising p-block elements and the respective element modification stable at ambient conditions [4, 8-19]. (bottom) Interatomic distances d vs. ionic radii for the selected compounds. Average distances of homonuclear Au-Au and heteronuclear Au-*E* (*E* – p-block element) are plotted separately.

5. Transmission electron microscopy and energy-dispersive X-ray spectroscopy

The compound was investigated using a JEOL 3000F analytical high-resolution transmission electron microscope with a field-emission electron source, operated at 300 kV. The microscope is equipped with a Gatan Orius camera (point-to-point resolution: 0.17 nm in TEM mode). The energy dispersive X-ray spectroscopy analysis was performed with an Oxford X-MAX XEDS system with an 80 mm^2 SDD detector. Imaging and analyses were conducted in a low-background beryllium double-tilt holder.

In-situ observation of changes of the samples upon heating in the transmission electron microscope was prevented by the instability of particles with appropriate thickness in the beam.

6. Physical properties

Magnetic susceptibility. The magnetic susceptibility measurement was conducted using a polycrystalline sample of cylindrical shape (34.31 mg) on a SQUID-based magnetic-properties measurement system magnetometer (MPMS, Quantum Design) between 3 and 400 K in an external field of 1000 Oe and at 300 K between 0 and 29000 Oe.

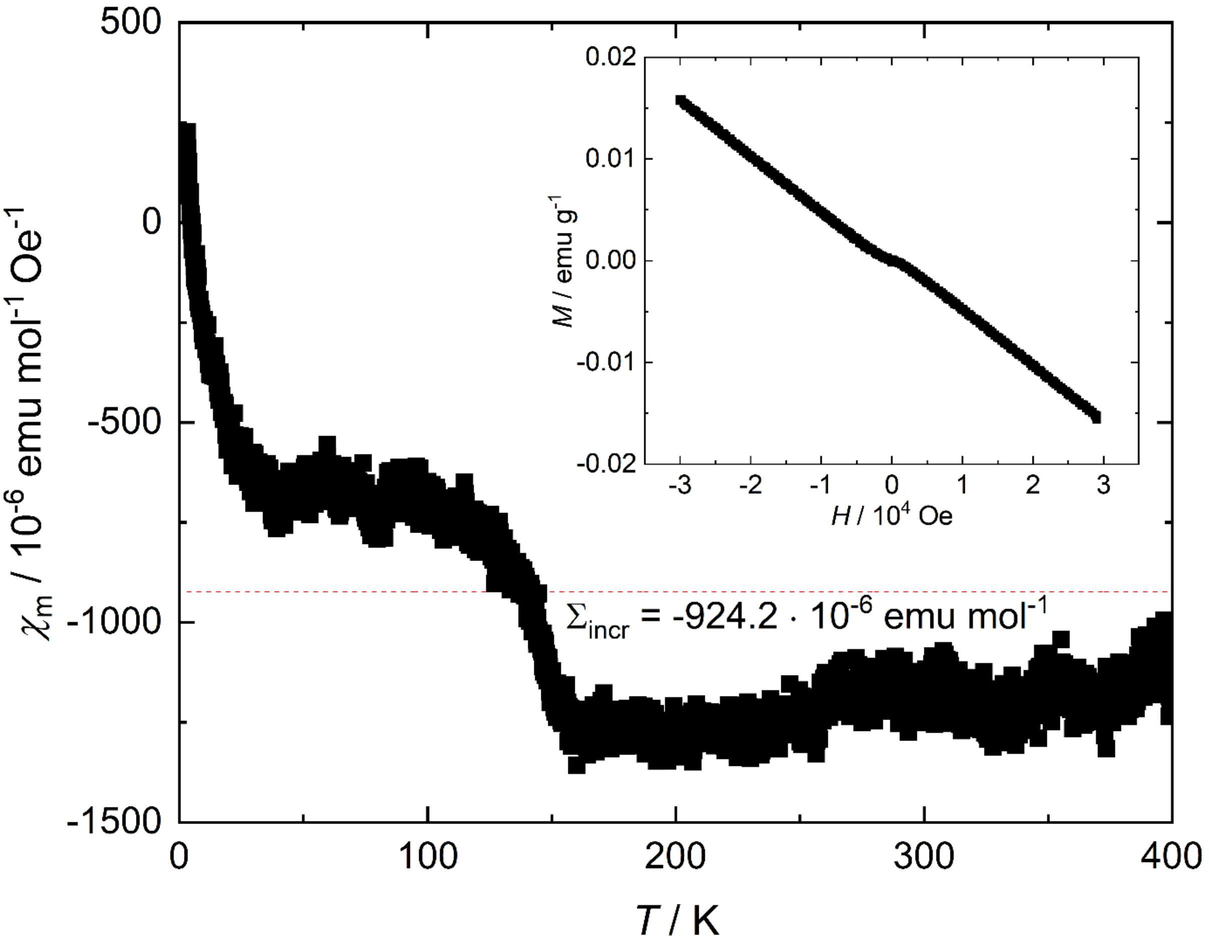

Figure S5. Molar susceptibility χ_M of Au_4Si in the temperature range of 2 to 400 K. The dashed line denotes the sum of the diamagnetic increments. Inset: Magnetization *M* as function of the magnetic field *H*.

The molar susceptibility χ_M (Figure S5) denotes diamagnetic behavior and remains constant between 260 and 400 K as well as between 160 and 260 K but at a slightly smaller value. The sum of the diamagnetic increments for Au and Si [24] amounts to $\Sigma_{incr} = -924.2 \times 10^{-6}$ emu mol^{-1}, which is in fair agreement with the measured values. Below 160 K, an upturn denoting minor paramagnetic impurity

is observed. The magnetization $M(H)$ (Figure S5, inset) at room temperature possesses a negative slope (-5.151(5) emu g^{-1} Oe^{-1}) in agreement with diamagnetic behavior.

Specific heat capacity. Heat capacity was measured from 2 to 400 K in magnetic fields up to 0.015 Oe in a Quantum Design Physical Property Measurement System.

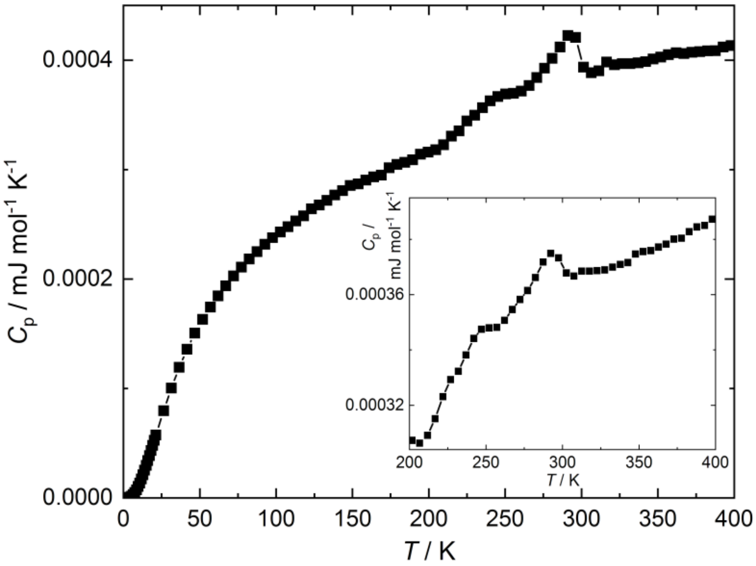

Figure S6. Specific heat C_p of Au_4Si, measured between 2 and 400 K. Inset: Specific heat C_p at 200 to 400K.

The specific heat C_p(T) of Au_4Si (Figure S6) shows a complex trend with a transition at T_{onset}=260 K and T_{mid}=295(5) K, taken from the first derivative of the specific heat C_p(T). DSC measurements in the temperature range from 185 to 310 K do not denote a phase transition (Figure S1, left, inset) and therefore, the observed slight variation in heat capacity near room temperature could be caused by the grease used to adhere the sample to the holder. The data are sufficiently described by the empirical formula $C_p=a+bT+cT^2+dT^3$ [25] with the constants derived from the fits listed in Table S4. The Debye temperature θ_D [25, 26] amounts to 254 K at low temperatures (2<T<210 K), 210 K at higher temperatures (200<T<400 K) and 289 K for the whole measured range (2<T<400 K), respectively (Table S4). The value for the higher temperature range is in sufficient agreement with the one previously reported for a mixture $Au_{81.4}Si_{18.6}$ (θ_D=220 K [2]), but is smaller than the experimental value derived from low-temperature data. The latter one is in good agreement with the value estimated from Neumann-Kopp's rule for eutectic mixtures [27] 259 K (at 0K [28]).

Table S4. Components of $C_p=aT+bT+cT^2+dT^3$ [mJ mol^{-1} K^{-1}] and calculated Debye temperature θ_D for Au_4Si.

Temperature range	a	b	c	d	θ_D
2-210 K	-0.28(2)	0.0047(1)	-2.5(1)	5.2(4)	254 K
200-400 K	-0.59(8)	0.0084(9)	-2.5(3)	2.5(3)	210 K
2-400 K	-0.006(4)	0.0032(1)	-9.6(7)	1.1(1)	289 K

7.1. Computational Procedures and Details

Electronic Structure Analysis: Structure optimization and the calculation of total energies, band energies, and density of states (DOS) distributions were performed for all Au_4Si structure variants using the Vienna ab initio Simulation Package (VASP) [29-32]. Calculations utilized the Projector Augmented Wave (PAW) [33,34] potentials and the Generalized Gradient Approximation (GGA) [35,36] in high precision mode. The computational parameters for these calculations are detailed in Table S5. Each structure was first geometrically optimized (Tables S6, S8-S10) before a single-point calculation was performed to generate the DOS distributions, electronic band energies, and total energies (Table S13). DOS curves were drawn with viewkel (a part of YAeHMOP) [37]. To better visualize the electronic structures, the degeneracy at the k-points of interest was broken by a slight modification of the atomic position of Si, as well as an alteration of the cell dimensions (Tables S7, S11-S12); these changes did not have a significant impact on the band structures generated (Figure S7).

The GGA-DFT electronic structure was used as the basis for the calibration of a simple Hückel model for investigation with the reversed approximation Molecular Orbital method [38]. The projected DOS distributions and band energies were used as reference data for the refinement of the semi-empirical parameters of the Hückel model with the eHtuner program [39]. The basis set consists of Slater-type orbitals corresponding to the Si 3s, Si 3p, Au 6s, and Au 6p atomic orbitals, along with double-ζ functions for the Au 5d. With the resulting parameters (Table S14), the root-mean-squared deviation in the band energies between the Hückel model and the DFT results is 0.885 eV for all bands up to 1.0 eV above the Fermi energy.

Using this simple Hückel model, a calculation was carried out on a 3×3×3 supercell of the structure to map the crystal orbitals of multiple k-points onto the Γ-point. The Hückel Hamiltonian was then imported into MATLAB, where the sequences of raMO constructions of first the Au 5d atomic orbitals and then either the Si 3s and 3p orbitals or the Au 6s orbitals, corresponding to the Zintl-like electron configurations $[Au^{+}]_4Si^{4-}$ and $[Au^{-}]_4Si^{4+}$, respectively. The raMO reconstructions were significantly more successful for the $[Au^{+}]_4Si^{4-}$ configuration, with the vast majority of the targets being well-reproduced; the first set of Si 3s and 3p orbitals in the sequence are shown in Figures S11. For the $[Au^{-}]_4Si^{4+}$ case, the first Au 6s reconstructions yield functions qualitatively localized to the central atom and its nearby surroundings, but these later give way to functions that are spread through the supercell, indicating that the occupied crystal orbitals are not well represented by a set of Au 6s-based functions.

To examine the potential influence of spin-orbit coupling (SOC), electronic structure calculations were performed with LDA-DFT using the ABINIT [40-42] package and Hartwigsen–Geodecker–Hutter norm-conserving pseudopotentials [43] for each of the three Au_4Si structure types. The structures were optimized with a two-step approach: first, the unit cell was held fixed while the atomic positions were optimized, then all parameters were relaxed simultaneously. Once the structures were optimized, DOS calculations were performed, both with and without SOC (Figures S8-S10). The features of the DOS near the Fermi energy are largely unchanged by the introduction of SOC.

Table S5. Computational parameters for VASP calculations.

Structure	Pseudopotential	Energy Cut-off (eV)	k-point Mesh
Au_4Si ($PtHg_4$-type)	GGA-PAW	306.8	12x12x12
Au_4Si (Distorted $PtHg_4$-type)	GGA-PAW	306.8	9x9x9
Au_4Si (Experimental Superstructure)	GGA-PAW	306.8	3×3×3

Table S6. Cell parameters for optimized geometries (GGA-PAW, VASP).

Structure	*a*	*b*	*c*	*α*	*β*	*Γ*
Au_4Si ($PtHg_4$-type)	5.870	5.870	5.870	90	90	90
Au_4Si (Distorted $PtHg_4$-type)	5.726	5.726	5.633	90	90	90
Au_4Si (Experimental Superstructure)	5.626	24.664	7.977	90	90	90

Table S7. Cell parameters for symmetry-broken geometries (GGA-PAW, VASP).

Structure	*a*	*b*	*c*	*α*	*β*	*Γ*
Au_4Si ($PtHg_4$-type)	5.870	5.880	5.860	90	90	90
Au_4Si (Distorted $PtHg_4$-type)	5.722	5.721	5.632	90	89.08	89.93

Table S8. Optimized atomic coordinates for Au_4Si in the $PtHg_4$ type (GGA-PAW, VASP).

Element	*x*	*y*	*z*
Si	0.0000	0.0000	0.0000
Si	0.5000	0.5000	0.5000
Au	0.2500	0.2500	0.2500
Au	0.7500	0.7500	0.7500
Au	0.7500	0.7500	0.2500
Au	0.2500	0.2500	0.7500
Au	0.7500	0.2500	0.7500
Au	0.2500	0.7500	0.2500
Au	0.2500	0.7500	0.7500
Au	0.7500	0.2500	0.2500

Table S9. Optimized atomic coordinates for Au_4Si in the distorted $PtHg_4$ type (GGA-PAW, VASP).

Element	*x*	*y*	*z*
Si	0.0000	0.0000	0.0000
Si	0.5000	0.5000	0.5000
Au	0.2962	0.2962	0.8409
Au	0.7038	0.7038	0.8409
Au	0.2962	0.7038	0.1591
Au	0.7038	0.2962	0.1591
Au	0.7962	0.7962	0.3409
Au	0.2038	0.2038	0.3409
Au	0.7962	0.2038	0.6591
Au	0.2038	0.7962	0.6591

Table S10. Optimized atomic coordinates for the experimental structure of Au_4Si (GGA-PAW, VASP).

Element	*x*	*y*	*z*
Si	0.9942	0.1666	0.5019
Si	0.9942	0.8334	0.4981
Si	0.9942	0.3334	0.0019
Si	0.9942	0.6666	0.9981
Si	0.4942	0.6666	0.5019
Si	0.4942	0.3333	0.4981
Si	0.4942	0.8334	0.0019
Si	0.4942	0.1666	0.9981
Si	0.9946	0.0000	0.0000
Si	0.9946	0.5000	0.5000
Si	0.4946	0.5000	0.0000
Si	0.4946	0.0000	0.5000
Au	0.1628	0.2357	0.9992
Au	0.1628	0.7643	0.0007
Au	0.1628	0.2643	0.4992
Au	0.1628	0.7357	0.5008
Au	0.6628	0.7357	0.9992
Au	0.6628	0.2643	0.0008
Au	0.6628	0.7643	0.4992
Au	0.6628	0.2357	0.5008
Au	0.8416	0.1682	0.1996
Au	0.8416	0.8318	0.8004
Au	0.8416	0.3318	0.6996
Au	0.8416	0.6682	0.3004
Au	0.3416	0.6682	0.1996
Au	0.3416	0.3318	0.8004
Au	0.3416	0.8318	0.6996
Au	0.3416	0.1682	0.3004
Au	0.8460	0.1649	0.8012
Au	0.8460	0.8351	0.1988
Au	0.8460	0.3351	0.3012
Au	0.8460	0.6649	0.6988
Au	0.3460	0.6649	0.8012
Au	0.3460	0.3351	0.1988
Au	0.3460	0.8351	0.3012
Au	0.3460	0.1649	0.6988
Au	0.1631	0.0976	0.0097
Au	0.1631	0.9024	0.9903

Au	0.1631	0.4024	0.5097
Au	0.1631	0.5976	0.4903
Au	0.6631	0.5976	0.0097
Au	0.6631	0.4024	0.9902
Au	0.6631	0.9024	0.5097
Au	0.6631	0.0976	0.4903
Au	0.6627	0.0690	0.0105
Au	0.6627	0.9310	0.9895
Au	0.6627	0.4310	0.5105
Au	0.6627	0.5690	0.4895
Au	0.1627	0.5690	0.0105
Au	0.1627	0.4310	0.9895
Au	0.1627	0.9310	0.5105
Au	0.1627	0.0690	0.4895
Au	0.3436	0.0034	0.8005
Au	0.3436	0.9966	0.1995
Au	0.3436	0.4966	0.3005
Au	0.3436	0.5034	0.6995
Au	0.8436	0.5034	0.8005
Au	0.8436	0.4966	0.1995
Au	0.8436	0.9966	0.3005
Au	0.8436	0.0034	0.6995

Table S11. Atomic coordinates for Au_4Si in the symmetry-broken $PtHg_4$ type (GGA-PAW, VASP).

Element	*X*	*Y*	*z*
Si	0.0000	0.0000	0.0000
Si	0.5010	0.5010	0.5000
Au	0.2500	0.2500	0.2500
Au	0.7500	0.7500	0.7500
Au	0.7500	0.7500	0.2500
Au	0.2500	0.2500	0.7500
Au	0.7500	0.2500	0.7500
Au	0.2500	0.7500	0.2500
Au	0.2500	0.7500	0.7500
Au	0.7500	0.2500	0.2500

Table S12. Atomic coordinates for Au_4Si in the symmetry-broken distorted $PtHg_4$ type (GGA-PAW, VASP).

Element	x	Y	z
Si	0.0000	0.0000	0.0000
Si	0.5000	0.5018	0.5000
Au	0.2971	0.2960	0.8403
Au	0.7049	0.7034	0.8408
Au	0.2950	0.7034	0.1594
Au	0.7029	0.2960	0.1599
Au	0.7971	0.7960	0.3404
Au	0.2049	0.2033	0.3409
Au	0.7951	0.2034	0.6594
Au	0.2030	0.7959	0.6599

Table S13. Total Energies (GGA-PAW, VASP).

Structure	Energy per Formula Unit (eV)
Au_4Si in $PtHg_4$ type	-18.037
Au_4Si in distorted $PtHg_4$ type	-18.420
Au_4Si, Experimental Superstructure	-18.434
Au_4Si in symmetry-broken $PtHg_4$ type	-18.037
Au_4Si in symmetry broken distorted $PtHg_4$ type	-18.419

Table S14. DFT-Calibrated Hückel Parameters for Au_4Si in the distorted $PtHg_4$ type.

Element	Orbital	H_{ii} (eV)	c_1	ζ_1	c_2	ζ_2
Si	Si 3s	-8.491		2.2396		
	Si 3p	-4.131		1.8960		
Au	Au 6s	-5.208		2.7885		
	Au 6p	-1.858		2.5652		
	Au 5d	-8.815	0.1411	6.1202	0.9202	3.0305

Table S15. Computational parameters for ABINIT calculations

Structure	XC Functional	Pseudopotentials	Energy Cutoff (Ha)	k-point Mesh
Au_4Si ($PtHg_4$-type)	LDA	HGH	60.0	9x9x9
Au_4Si (Distorted $PtHg_4$-type)	LDA	HGH	40.0	9x9x9
Au_4Si (Experimental Superstructure)	LDA	HGH	40.0	6x6x4

Table S16. Total Energies for ABINIT calculations

Structure	Energy per Formula Unit (Ha)
Au_4Si in $PtHg_4$ type	-137.177
Au_4Si in distorted $PtHg_4$ type	-137.188
Au_4Si, Experimental Superstructure	-137.188
Au_4Si in $PtHg_4$ type with SOC	-137.258
Au_4Si in distorted $PtHg_4$ type with SOC	-137.270
Au_4Si, Experimental Superstructure with SOC	-137.270

Table S17. Cell parameters for ABINIT optimized geometries (distances in Bohr)

Structure	a	b	c	α	β	Γ
Au_4Si ($PtHg_4$-type)	9.368	9.368	9.368	109.47	109.47	109.47
Au_4Si (Distorted $PtHg_4$-type)	9.143	9.143	9.143	109.10	109.10	110.22
Au_4Si (Experimental Superstructure)	22.949	22.949	14.944	90	90	153.85

Table S18. ABINIT Optimized atomic coordinates for Au_4Si in the $PtHg_4$ type

Element	x	y	z
Si	0.0000	0.0000	0.0000
Au	0.5000	0.5000	0.5000
Au	0.0000	0.0000	0.5000
Au	0.0000	0.5000	0.0000
Au	0.5000	0.0000	0.0000

Table S19. ABINIT Optimized atomic coordinates for Au_4Si in the distorted $PtHg_4$ type

Element	*x*	*y*	*z*
Si	0.0000	0.0000	0.0000
Au	0.1362	0.1362	0.5942
Au	0.4560	0.8638	0.0000
Au	0.5420	0.5420	0.4058
Au	0.8638	0.4580	0.0000

Table S20. ABINIT Optimized atomic coordinates for the experimental structure of Au_4Si

Element	*x*	*y*	*z*
Si	0.8378	0.1723	0.5090
Si	0.0037	0.0037	0.0000
Si	0.1723	0.8378	0.4910
Si	0.6723	0.3378	0.0090
Si	0.5037	0.5037	0.5000
Si	0.3378	0.6723	0.9910
Au	0.9165	0.3797	0.9775
Au	0.6605	0.0139	0.2060
Au	0.7011	0.9971	0.7972
Au	0.0672	0.2611	0.0530
Au	0.5931	0.7254	0.0593
Au	0.3259	0.3673	0.7952
Au	0.3797	0.9165	0.0225
Au	0.0139	0.6605	0.7940
Au	0.9971	0.7011	0.2028
Au	0.2611	0.0672	0.9470
Au	0.7254	0.5931	0.9407
Au	0.3673	0.3259	0.2048
Au	0.8797	0.4165	0.4775
Au	0.5139	0.1605	0.7060
Au	0.4971	0.2011	0.2972
Au	0.7611	0.5672	0.5530
Au	0.2254	0.0931	0.5593
Au	0.8673	0.8259	0.2952
Au	0.4165	0.8797	0.5225
Au	0.1605	0.5139	0.2940
Au	0.2011	0.4971	0.7028
Au	0.5672	0.7611	0.4470
Au	0.0931	0.2254	0.4407
Au	0.8259	0.8673	0.7048

7.2. Band Structures and Results of Calculations with Spin-Orbit Coupling.

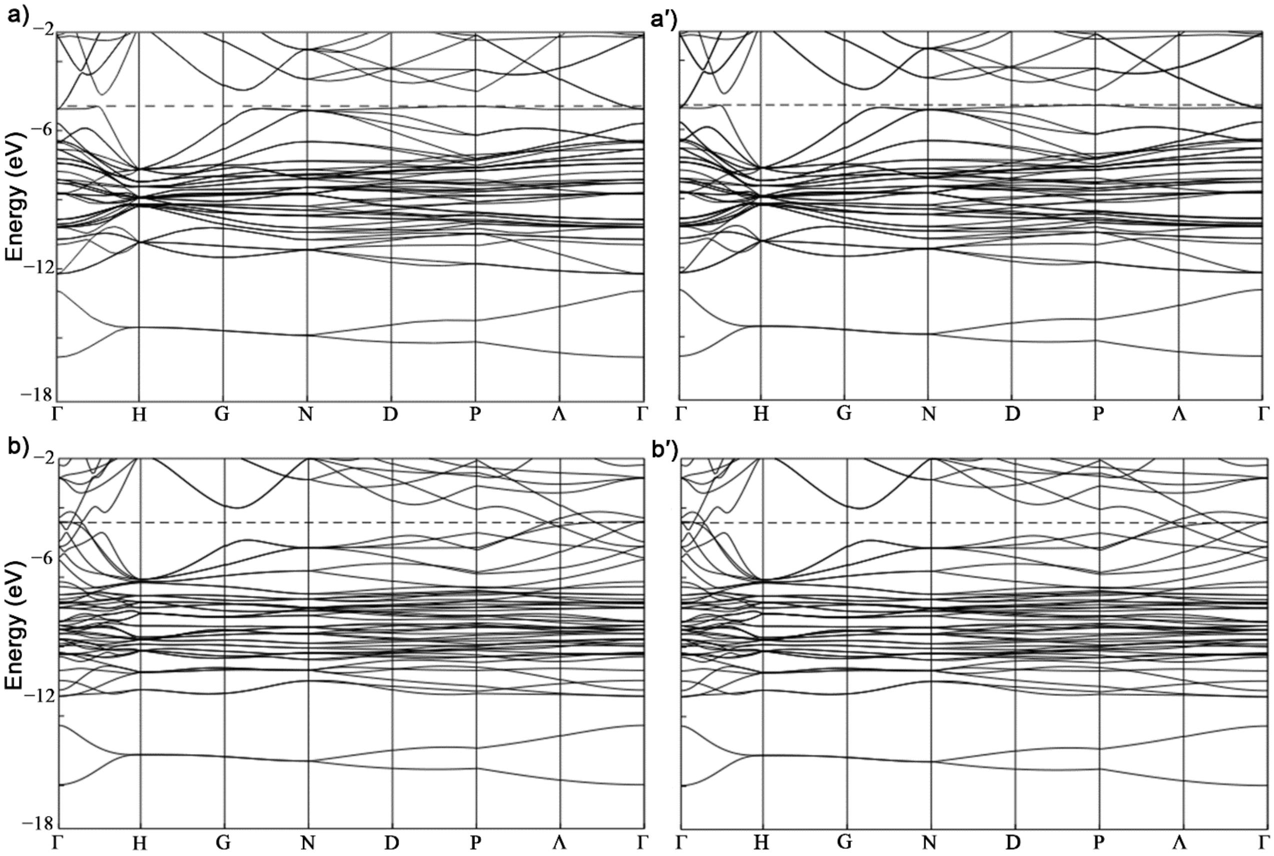

Figure S7. Comparison of band plot of degenerate structures to the band plot of the non-degenerate structures (a) Au_4Si in the $PtHg_4$ type and (b) Au_4Si in the distorted version of the $PtHg_4$ type. (a′) and (b′) give the corresponding band structures for phases in which the symmetry is weakly broken to remove band degeneracies.

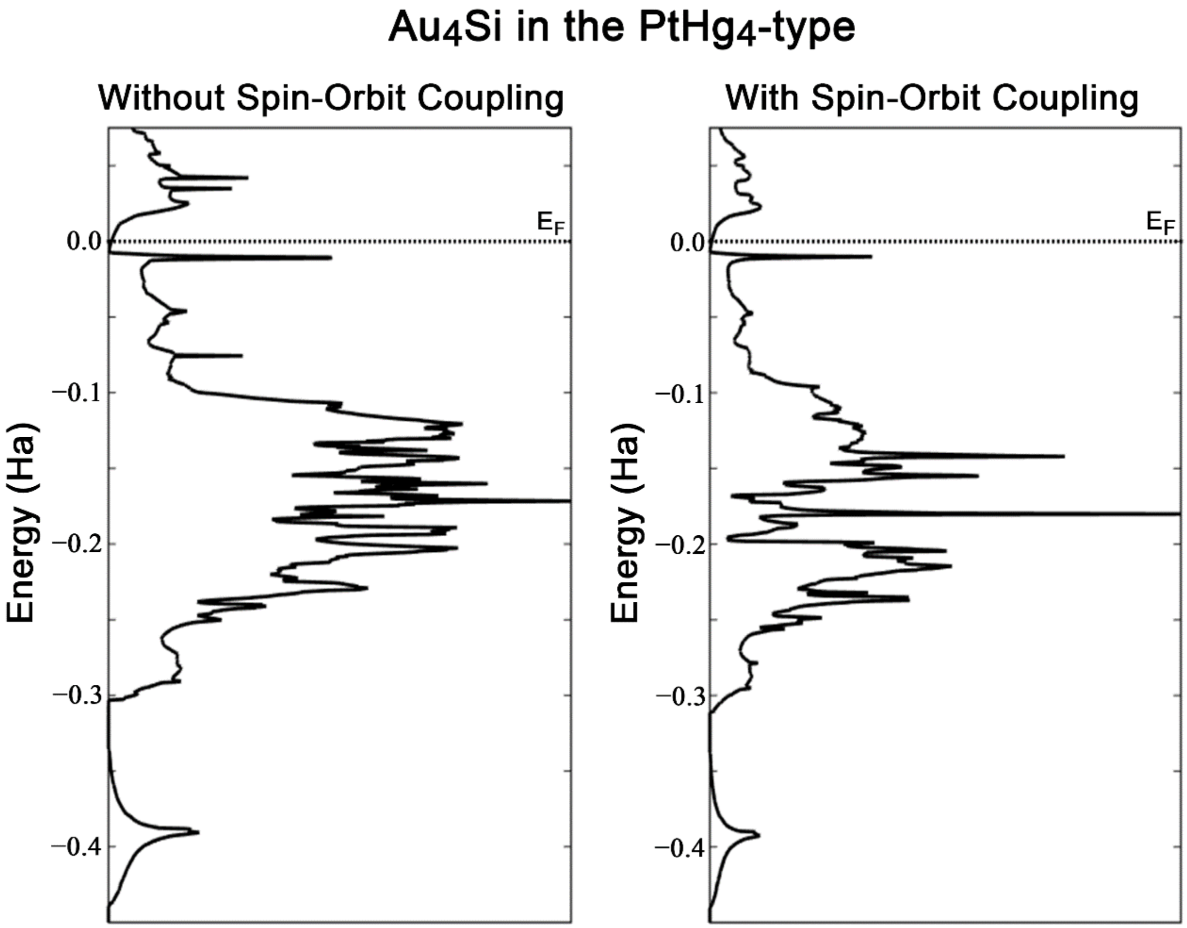

Figure S8. Electronic DOS curves calculated in ABINIT for Au_4Si in the $PtHg_4$-type without (left) and with (right) spin-orbit coupling.

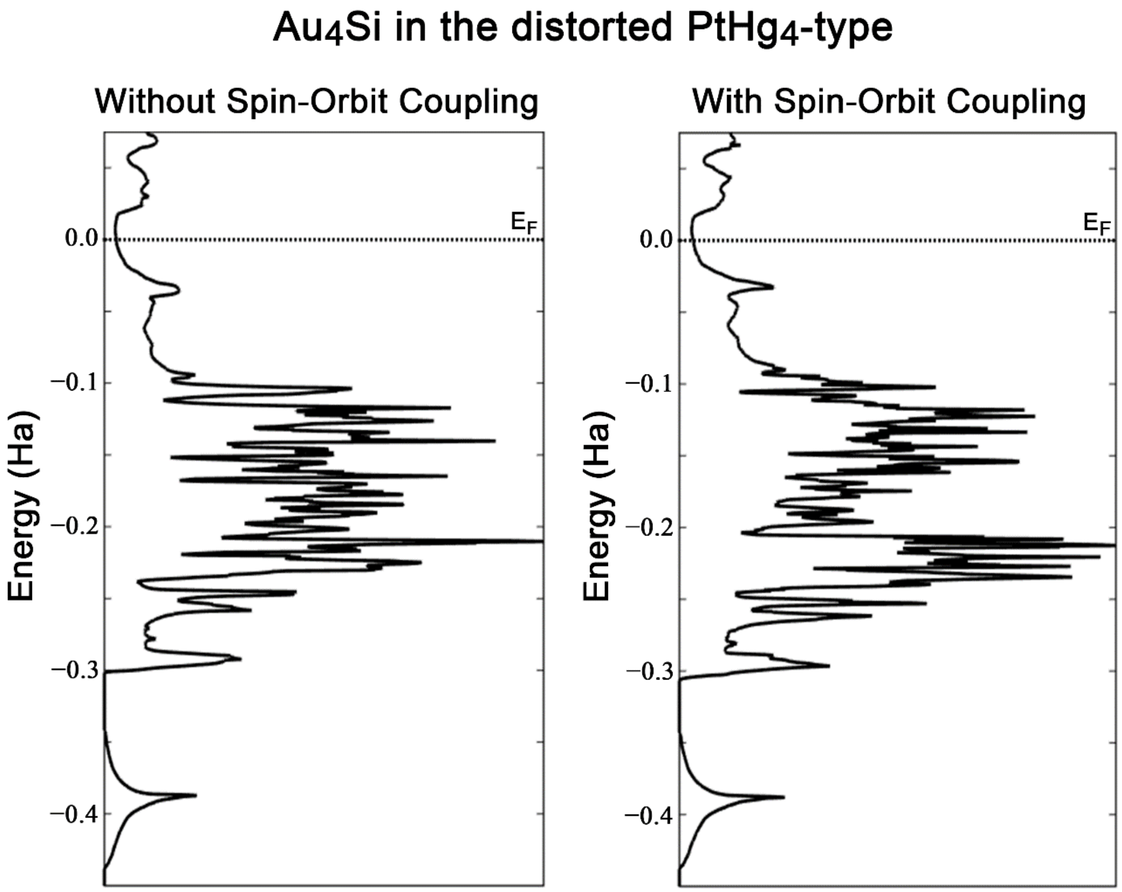

Figure S9. Electronic DOS curves calculated in ABINIT for Au_4Si in the distorted $PtHg_4$-type without (left) and with (right) spin-orbit coupling

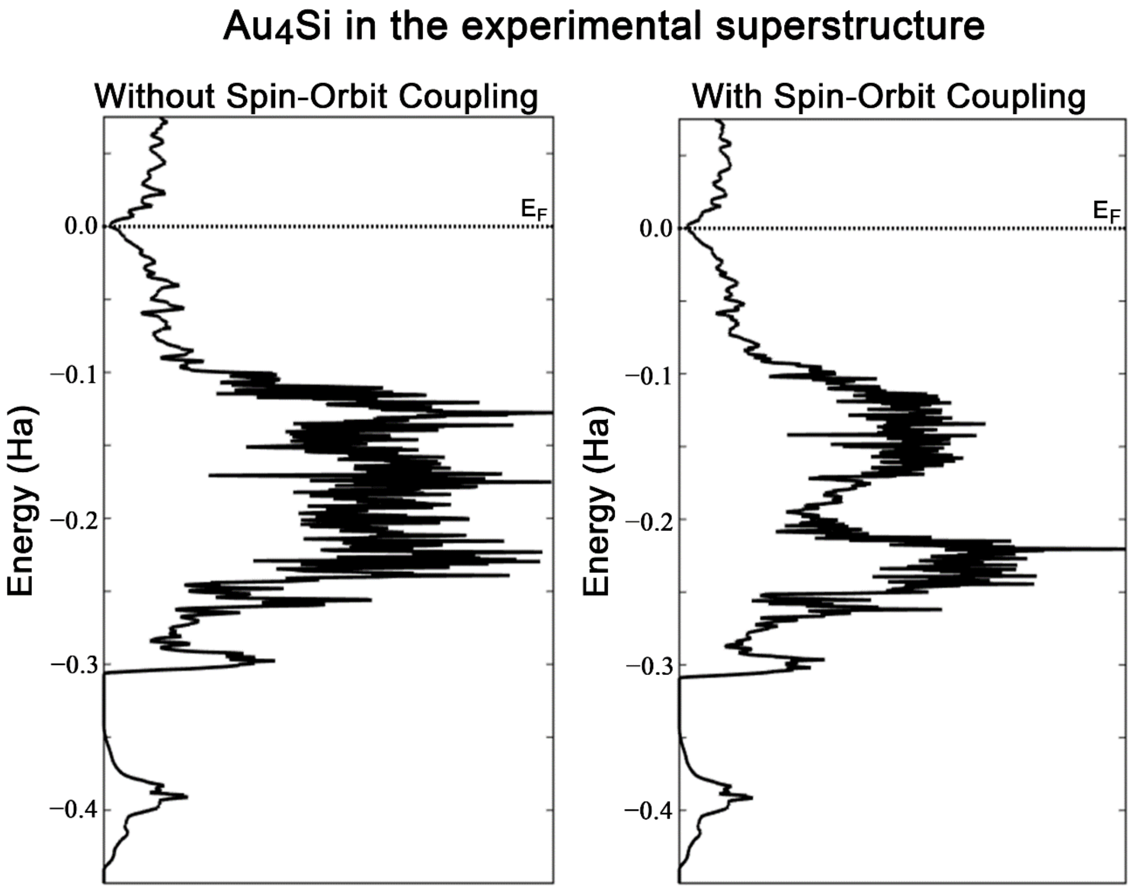

Figure S10. Electronic DOS curves calculated in ABINIT for Au_4Si in the experimental superstructure without (left) and with (right) spin-orbit coupling

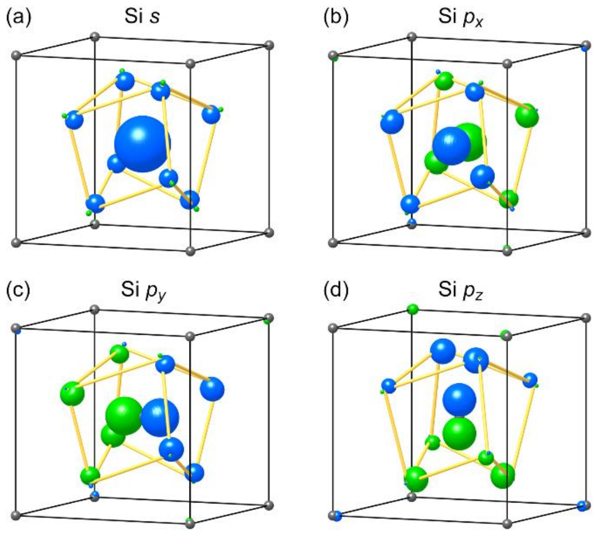

Figure S11. raMO constructions of the (a) 3s and (b)-(d) 3p orbitals of a Si atom in the distorted $PtHg_4$-type model for Au_4Si. The contents of one $PtHg_4$-type cell within the 3×3×3 supercell are shown.

References

[1] Kurtuldu, G. & Löffler, J.F. Multistep Crystallization and Melting Pathways in the Free-Energy Landscape of a Au–Si Eutectic Alloy. *Adv. Sci.*, **2020**, *7*, 1903544.

[2] Chen, H.S. & Turnbull, D. Thermal Properties of Gold-Silicon Binary Alloy near the Eutectic Composition, *J. Appl. Phys*. **1967**, *38*(9), 3646-3650.

[3] Buechler, H. & Range, K.J. Zur Kenntnis des beta-Mangan-Typs: Hochdrucksynthese und Strukturverfeinerung von $AlAu_4$. *J. Less Common Met*. **1990**, *161*, 347-354.

[4] Buechler, H. & Range, K.J. Hochdrucksynthese und Kristallstruktur von Al_3Au_8. *J. Less Common Met*. **1989**, 154(2), 251-260.

[5] Agilent. CrysAlis PRO (Agilent Technologies Ltd.: Yarnton, Oxfordshire, England, 2014).

[6] Palatinus, L.; Chapuis, G. SUPERFLIP - a computer program for the solution of crystal structures by charge flipping in arbitrary dimensions. *J. Appl. Crystallogr.* **2007**, *40*, 786– 790.

[7] Petříček, V.; Dusek, M.; Palatinus, L. Crystallographic Computing System JANA2006: General features. *Z. Kristallogr. - Cryst. Mater*. **2014**, *229* (5), 345– 352.

[8] Brandon, J.K.; Brezard, R.; Pearson, W.B.; Tozer, D.J.N. Gamma-brasses with I and P cells. Acta Crystallogr. B **1977**, *33*, 527-537.

[9] Puselj, M.; Schubert, K. Kristallstrukturen der Phasen Au_2Al(h), Au_2Al^{1-}(r) und Au_2Al^{1+}(r). *J. Less Common Met.* **1974**, *35*(2), 259-266.

[10] Frank, K. Kristallstruktur von Au_7Ga_2(h). *J. Less Common Met.* **1971**, *23*(1), 83-87.

[11] Chen, K.W.; Graf, D.; Besara, T.; Gallagher, A.; Kikugawa, N.; Balicas, L.; Siegrist, T.; Shekhter, A.; Baumbach, R.E. Temperature-pressure phase diagram of cubic Laves phase Au_2Pb. *Phys. Rev. B* **2016**, *93*(4), 045118-1-5.

[12] Schoop, Leslie M.; Xie, Lilia S.; Chen, Ru; Gibson, Quinn D.; Lapidus, Saul H.; Kimchi, Itamar; Hirschberger, Max; Haldolaarachchige, Neel; Ali, Mazhar N.; Belvin, Carina A.; Liang, Tian; Neaton, Jeffrey B.; Vishwanath, Ashvin; Cava, R.J. Dirac metal to topological metal transition at a structural phase change in Au_2Pb and prediction of Z2 topology for the superconductor. *Phys. Rev. B* **2015**, *91*(21) 214517-1-6.

[13] Osada, K.; Yamaguchi, S.; Hirabayashi, M. An ordered stucture of Au_5Sn. *Trans. Jpn. Inst. Met*. **1974**, *15*, 256-260.

[14] Jurriaanse, T. The crystal structure of Au_2Bi. *Z. Kristallogr.* **1935**, *90*, 322-329.

[15] Hirsch, H., de Cugnac, A., Gadet, M.-C., Pouradier, J. Cristallographie du sulfure aureux. *C. r. séances Acad. sci., Sér. 3, Sci. vie*. **1966**, *263*, 1328-1330.

[16] Puselj, M.; Schubert, K. Kristalstrukturen von Au_9In_4 (h) und Au_7In_3. *J. Less Common Met.* **1975**, 41, 33-44.

[17] Jandali, M.Z.; Rajasekharan, T.; Schubert, K. Crystal structure of $Au_{10}In_3$. *Z. Metallkd.* **1982**, *73*, 463-467.

[18] Puselj, M.; Schubert, K. Kristallstruktur von Au_2Ga. *J. Less Common Met.* **1974**, *38*, 83-90.

[19] Straumanis, M.E. Neubestimmung der Gitterparameter, Dichten und thermischen Ausdehnungskoeffizienten von Silber und Gold, und Vollkommenheit der Struktur. Monatsh. Chem. **1971**, *102*, 1377-1386.

[20] Kresse, G.; Furthmüller, J. Efficient iterative schemes for ab initio total-energy calculations using a plane-wave basis set. Phys. Rev. B: Condens. Matter **1996**, 54, 11169– 11186,

[21] Kresse, G.; Furthmüller, J. Efficiency of ab-initio total energy calculations for metals and semiconductors using a plane-wave basis set. Comput. Mater. Sci. **1996**, 6, 15– 50,

[22] Blöchl, P. E. Projector augmented-wave method. Phys. Rev. B 1994, 50, 17953– 17979,

[23] Kresse, G.; Joubert, D. From ultrasoft pseudopotentials to the projector augmented-wave method. Phys. Rev. B 1999, 59, 1758– 1775,

[24] Haynes, W. M. *CRC Handbook of Chemistry and Physics* (CRC Press, 2012).

[25] Tinkham, M. *Introduction to Superconductivity* (McGraw–Hill, 1996).

[26] Kittel, C. *Introduction to Solid State Physics* (Wiley, 2005).

[27] Leitner, J., Voňka, P., Sedmidubský, D., Svoboda, P. Application of Neumann–Kopp rule for the estimation of heat capacity of mixed oxides, *Thermochim. Acta*, **497**(1–2), 7-13 (2010).

[28] Stewart, G. R. Measurement of Low-Temperature Specific Heat. *Rev. Sci. Instrum*. **54**(1), 1–11 (1983).

[29] Kresse, G.; Hafner, J. Ab Initio Molecular Dynamics for Liquid Metals. *Phys. Rev. B* **1993**, *47* (1), 558–561.

[30] Kresse, G.; Furthmüller, J. Efficient Iterative Schemes for Ab Initio Total-Energy Calculations Using a Plane-Wave Basis Set. *Phys. Rev. B - Condens. Matter Mater. Phys.* **1996**, *54* (16), 11169–11186.

[31] Kresse, G.; Furthmüller, J. Efficiency of Ab-Initio Total Energy Calculations for Metals and Semiconductors Using a Plane-Wave Basis Set. *Comput. Mater. Sci.* **1996**, *6* (1), 15–50.

[32] Kresse, G.; Hafner, J. Ab Initio Molecular-Dynamics Simulation of the Liquid-Metalamorphous-Semiconductor Transition in Germanium. *Phys. Rev. B* **1994**, *49* (20), 14251–14269.

[33] Blöchl, P. E. Projector Augmented-Wave Method. *Phys. Rev. B* **1994**, *50* (24), 17953–17979.

[34] Joubert, D. From Ultrasoft Pseudopotentials to the Projector Augmented-Wave Method. *Phys. Rev. B* -

Condens. Matter Mater. Phys. **1999**, *59* (3), 1758–1775.

[35] Perdew, J. P.; Chevary, J. A.; Vosko, S. H.; Jackson, K. A.; Pederson, M. R.; Singh, D. J.; Fiolhais, C. Atoms, Molecules, Solids, and Surfaces: Applications of the Generalized Gradient Approximation for Exchange and Correlation. *Phys. Rev. B* **1992**, *46* (11), 6671–6687.

[36] Perdew, J. P.; Chevary, J. A.; Vosko, S. H.; Jackson, K. A.; Pederson, M. R.; Singh, D. J.; Fiolhais, C. Erratum: Atoms, Molecules, Solids, and Surfaces: Applications of the Generalized Gradient Approximation for Exchange and Correlation (Physical Review B (1993) 48, 7, (4978)). *Physical Review B*. American Physical Society August 15, 1993, p 4978.

[37] Landrum, G.; Glassey, W. YAeHMOP: Yet Another extended Hückel Molecular Orbital Package. YAeHMOP is freely available via the Internet at URL: http://yaehmop.sourceforge.net/

[38] Yannello, V. J.; Kilduff, B. J.; Fredrickson, D. C. Isolobal Analogies in Intermetallics: The Reversed Approximation Mo Approach and Applications to CrGa4- and Ir3Ge 7-Type Phases. *Inorg. Chem.* **2014**, *53* (5), 2730–2741.

[39] Stacey, T. E.; Fredrickson, D. C. Perceiving Molecular Themes in the Structures and Bonding of Intermetallic Phases: The Role of Hückel Theory in an Ab Initio Era. *Dalton Transactions*. The Royal Society of Chemistry July 14, 2012, 7801–7813.

[40] Gonze, X.; Rignanese, G. M.; Verstraete, M.; Beuken, J. M.; Pouillon, Y.; Caracas, R.; Jollet, F.; Torrent, M.; Zerah, G.; Mikami, M.; Ghosez, P.; Veithen, M.; Raty, J. Y.; Olevano, V.; Bruneval, F.; Reining, L.; Godby, R.; Onida, G.; Hamann, D. R.; Allan, D. C. A Brief Introduction to the ABINIT Software Package. *Zeitschrift fur Krist.* **2005**, *220* (5–6), 558–562.

[41] Gonze, X.; Beuken, J. M.; Caracas, R.; Detraux, F.; Fuchs, M.; Rignanese, G. M.; Sindic, L.; Verstraete, M.; Zerah, G.; Jollet, F.; Torrent, M.; Roy, A.; Mikami, M.; Ghosez, P.; Raty, J. Y.; Allan, D. C. First-Principles Computation of Material Properties: The ABINIT Software Project. *Comput. Mater. Sci.* **2002**, *25* (3), 478–492.

[42] Gonze, X.; Amadon, B.; Anglade, P. M.; Beuken, J. M.; Bottin, F.; Boulanger, P.; Bruneval, F.; Caliste, D.; Caracas, R.; Côté, M.; Deutsch, T.; Genovese, L.; Ghosez, P.; Giantomassi, M.; Goedecker, S.; Hamann, D. R.; Hermet, P.; Jollet, F.; Jomard, G.; Leroux, S.; Mancini, M.; Mazevet, S.; Oliveira, M. J. T.; Onida, G.; Pouillon, Y.; Rangel, T.; Rignanese, G. M.; Sangalli, D.; Shaltaf, R.; Torrent, M.; Verstraete, M. J.; Zerah, G.; Zwanziger, J. W. ABINIT: First-Principles Approach to Material and Nanosystem Properties. *Comput. Phys. Commun.* **2009**, *180* (12), 2582–2615.

[43] Hartwigsen, C.; Goedecker, S.; Hutter, J. Relativistic Separable Dual-Space Gaussian Pseudopotentials from H to Rn. *Phys. Rev. B - Condens. Matter Mater. Phys.* **1998**, *58* (7), 3641–3662.
